# The Mexican experience in monitoring and evaluation of public policies addressing social determinants of health

**DOI:** 10.3402/gha.v9.29030

**Published:** 2016-02-23

**Authors:** Adolfo Martinez Valle

**Affiliations:** Economic Analysis Unit, Ministry of Health, Mexico City, Mexico

**Keywords:** monitoring, evaluation, health inequities, health inequalities, public policies, social determinants of health, Mexico

## Abstract

Monitoring and evaluation (M&E) have gradually become important and regular components of the policy-making process in Mexico since, and even before, the World Health Organization (WHO) Commission on Social Determinants of Health (CSDH) called for interventions and policies aimed at tackling the social determinants of health (SDH). This paper presents two case studies to show how public policies addressing the SDH have been monitored and evaluated in Mexico using reliable, valid, and complete information, which is not regularly available. Prospera, for example, evaluated programs seeking to improve the living conditions of families in extreme poverty in terms of direct effects on health, nutrition, education and income. Monitoring of Prospera's implementation has also helped policy-makers identify windows of opportunity to improve the design and operation of the program. Seguro Popular has monitored the reduction of health inequalities and inequities evaluated the positive effects of providing financial protection to its target population. Useful and sound evidence of the impact of programs such as Progresa and Seguro Popular plus legal mandates, and a regulatory evaluation agency, the National Council for Social Development Policy Evaluation, have been fundamental to institutionalizing M&E in Mexico. The Mexican experience may provide useful lessons for other countries facing the challenge of institutionalizing the M&E of public policy processes to assess the effects of SDH as recommended by the WHO CSDH.

## Introduction

A decade ago, the World Health Organization (WHO) Commission on Social Determinants of Health (CSDH) called for interventions and policies aimed at the social determinants of health (SDH). One of its key recommendations was to measure health inequality and inequity while evaluating the impact of actions addressing SDH (1). Several public policies tackling health inequities, that result in part by unfair societal factors, have been implemented in Mexico – since and even before the CSDH was established. Most of these public policies aimed to fight poverty and/or protect the income of nearly half of Mexico's population. However only a few of them, have been rigorously monitored and evaluated. We focus herein only on public policies that have explicitly addressed “health-influencing experiences that result from the unequal distribution of political power, income, and resources, the resulting disparity in daily living circumstances, such as access to healthcare and education and living and working conditions, and the lower potential of socioeconomically disadvantaged individuals to lead prosperous lives” (1).

The absence of rigorous monitoring and evaluation (M&E) is explained in part by the fact that reliable, valid, and complete data are not regularly available. Case studies are used here to show how national policies in Mexico have been monitored and evaluated using available empirical evidence. Together with the creation of government agencies and instruments, and some fostering by WHO CSDH, Mexico has begun to institutionalise M&E.

## M&E of policies tackling SDH in Mexico

For this study we selected two national public policies that tackled health inequities associated with the SDH and implemented M&E. The first is the conditional cash transfer program, now called Prospera.^1^ The program was implemented in 1997 to ameliorate the extreme poverty in which a quarter of Mexico's population had been living for the previous two decades. Prospera has systematically demonstrated direct effects on health (2–7) and nutrition (8–11) outcomes, and on important social determinants such as education (12–14). The program is regarded as setting international standards for social policy evaluation (15, 16).

**Fig. 1:**
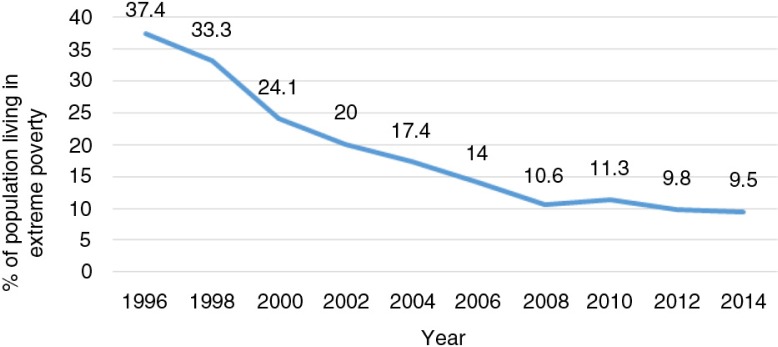
Extreme poverty evolution in Mexico, 1996–2014. From Ref. (18).

The monitoring of Prospera's implementation has also helped policy-makers identify windows of opportunity to improve its design and operation (17). One of the main indicators used to monitor its performance is the percentage of people living in extreme poverty. This has been regularly measured every 2 years through the national income and expenditure surveys were introduced. Since Prospera was introduced, as this percentage has been gradually diminishing (Fig. 1). Although this reduction cannot be attributed only to the Prospera program, there is evidence indicating its effect on alleviating, or at least containing, the growth of extreme poverty (15, 19–24).

**Fig. 2:**
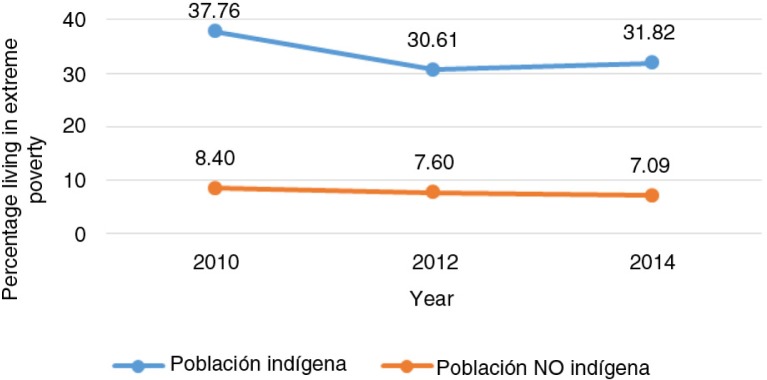
Percentage of population living in extreme poverty by indigenous condition, 2010–2014. From Ref. (25).

This indicator – the percentage of the population living in extreme poverty – does not, however, identify who the most socially disadvantaged individuals living in extreme poverty are. Figure 2 shows that most people living in extreme poverty are indigenous.^2^ This highlights the importance of developing specific strategies to address this important inequity.

The more recent program, called Seguro Popular de Salud (SPS), is a national insurance scheme designed in 2002 and implemented since that time to protect the income of the population not covered by social security. It provides explicit health insurance coverage for a fairly comprehensive benefit package. Evaluations of SPS have provided empirical evidence of the positive effects of this financial protection, especially for the low-income population (26–32). Evidence shows a reduction in the inequitable allocation of resources between the insured and the uninsured population (33, 34).

**Fig. 3:**
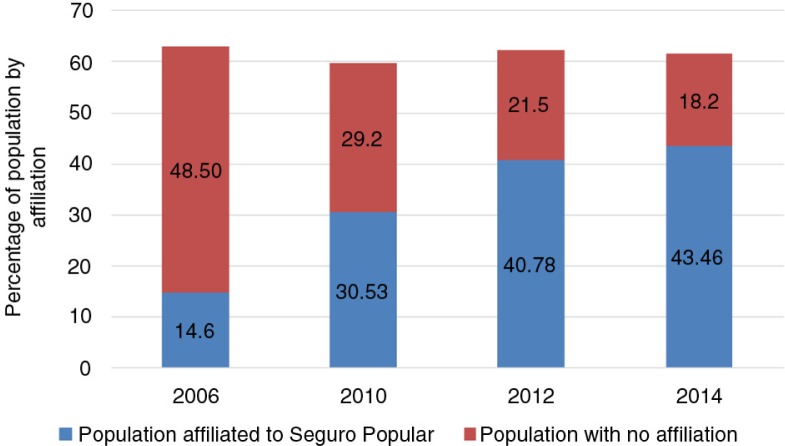
Percentage of population by insurance coverage, 2006–2014. From Refs. (18, 37).

**Fig. 4:**
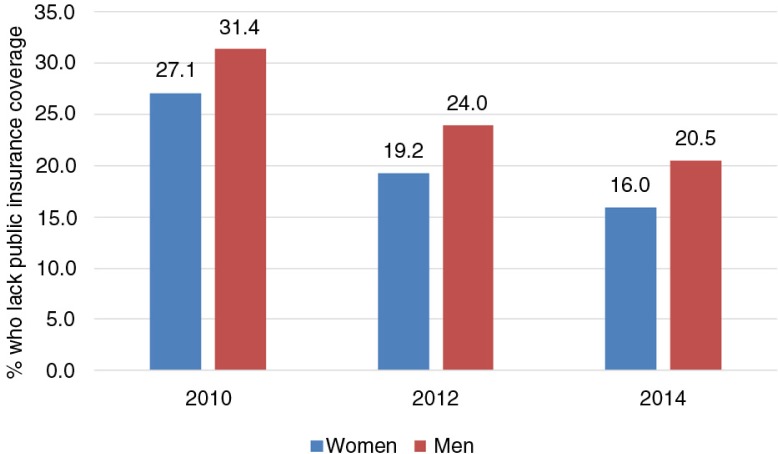
Percentage who lack insurance coverage by sex, 2010–2014. From Ref. (18).

Seguro Popular has been monitored using health insurance coverage as the main indicator. National health surveys and national income and expenditure surveys were used to gather data. Both datasets have shown increasing coverage of the affiliated population (Fig. 3). When disaggregated by sex, the population not covered by a public insurance scheme shows how men have a lower coverage than women (Fig. 4).

Despite this progress, full access to public health care in Mexico has not yet been achieved. Users keep seeking private services to take care of their health problems resulting in still relatively high out-of-pocket payments. Out-of-pocket payments have diminished from 51.7% of total health expenditures in 2004 to only 44% in 2013 (32).

## Institutionalization of M&E

M&E has gradually become an important component of public policy-making in Mexico, and Prospera has set the example. Soon after the implementation of Prospera, Congress mandated that all programs with operating rules^3^ had to be evaluated annually by external evaluators. The mandate covered approximately 25–30% of the federal budget for programs. The number of evaluations jumped from single digits to over a hundred in 2001 and in subsequent years (35). Seguro Popular was one of those programs.

The Social Development Law and the creation of the National Council for the Evaluation of Social Policy (CONEVAL) in 2004 further institutionalized evaluation. CONEVAL was designed as an autonomous institution whose mission was to measure poverty reduction results nationally and coordinate evaluation of social programs by the federal government. Its independence and technical capacities^4^ as well as the mandate allowed it to advance construction of the social sector M&E system. The experience of CONEVAL has become a benchmark for other developing countries undertaking M&E reforms (35).

Although there is more evaluation to be done, still very little of that information has been effectively used. There was little awareness then of the role that evaluation plays in improving government programs. Although by law, every health-related policy should undergo some form of evaluation, evaluation guidelines have not always been followed. They are difficult to enforce. Evaluation activity has also lacked the incentives and institutional arrangements to ensure use of the findings.

More recently, under National Health Programs since 2007, the health sector agenda has explicitly addressed persistent inequalities associated with the distribution of wealth and other socioeconomic factors, plus health system characteristics that shape the rules of financing and access to health services (36). Furthermore, in order to monitor progress toward the overall goal of achieving more inclusive development, indicators that address SDH have been included in the current government's National Health Program (37) (Table 1).

**Table 1:** Monitoring indicators of the National Health Program, 2013–2018

Indicator	Baseline (2012) (%)	Value (2014) (%)	Goal (2018) (%)
Percentage of the population without public health insurance	21.5	18.2	6.0
Percentage of the population covered by public insurance and using public health care services	53.8	63.3	80.0
Percentage of households of the lowest income quintile with catastrophic health care expenditures	4.6	4.5	3.5

From Ref. (36).

## Future challenges

Although institutional foundations are in place, important challenges to achieving full institutionalization of M&E remain in Mexico. The main challenge is the widespread use of M&E as a transparent policy-making practice. Transparency and accountability have fostered M&E of public policies in Mexico. However, the utility of these practices is yet to be fully recognized in policy-making circles. This challenge is closely linked to the following:

- Develop capacity building for evaluation at all levels of government to measure the effect of public policies on target populations.
- Foster the practice of monitoring in policy-making. Monitoring is still is not as developed as evaluation. Monitoring mostly relies on administrative data that are usually not as reliable and complete as the data used for longer-term in-depth evaluations that focus more on outcomes and impacts.
- Strengthen health information systems to provide disaggregated data by socioeconomic groups for monitoring health inequalities. This is essential for everyday decision-making, because government performance is strongly linked to services and products that need to be managed on a daily basis. Public policy practices must complement each other with equally rigorous methodologies.

In sum, M&E has gradually become an important and regular part of the policy-making process in Mexico. Both the production of useful and sound evidence on the impact of programs such as Prospera and Seguro Popular, as well as legal mandates, and strong institutions such as CONEVAL, have been critical in institutionalizing M&E. The Mexican experience may provide useful lessons for other countries wanting to assess the effects of SDH by institutionalizing, monitoring, and evaluating their own public policy processes.

## Footnotes

1 Prospera was originally named Progresa in 1997. It was then changed to Oportunidades in 2002 and to Prospera in 2014. These name changes have been principally because of different political parties being in power. The essential characteristics of a conditional cash transfer program have remained unchanged.

2 Indigenous are those individuals that either speak a native language or live in a household where a native language is spoken.

3 Rules of operation required every program that received federal budget to provide basic information about its design, objectives, performance indicators, target population, and procedures.

4 CONEVAL relies on an independent collegiate body made up of six academic councilors. These six individuals are democratically elected for a period of 4 years, and they are chosen from certified academic institutions. The councilors are involved in all of the agency's decisions and the definition and review of evaluation projects. They also provide general guidance on the administrative direction of the institution and play an important role in the methodologies for poverty measurement.
